# Mitochondrial stress response and myogenic differentiation

**DOI:** 10.3389/fcell.2024.1381417

**Published:** 2024-04-12

**Authors:** Fu Lin, Liankun Sun, Yu Zhang, Weinan Gao, Zihan Chen, Yanan Liu, Kai Tian, Xuyu Han, Ruize Liu, Yang Li, Luyan Shen

**Affiliations:** ^1^ Key Laboratory of Pathobiology, Department of Pathophysiology, Ministry of Education, College of Basic Medical Sciences, Jilin University, Changchun, China; ^2^ Experimental Teaching Center of Basic Medicine, College of Basic Medical Sciences, Jilin University, Changchun, China; ^3^ Clinical Medical College of Jilin University, The First Hospital of Jilin University, Changchun, China; ^4^ China Japan Union Hospital of Jilin University, Changchun, China; ^5^ Department of Physiology, College of Basic Medical Sciences, Jilin University, Changchun, China

**Keywords:** myogenic differentiation, ISR, UPRmt, mitochondrial biogenesis, mitochondrial fusion and fission, mitophagy, apoptosis

## Abstract

Regeneration and repair are prerequisites for maintaining effective function of skeletal muscle under high energy demands, and myogenic differentiation is one of the key steps in the regeneration and repair process. A striking feature of the process of myogenic differentiation is the alteration of mitochondria in number and function. Mitochondrial dysfunction can activate a number of transcriptional, translational and post-translational programmes and pathways to maintain cellular homeostasis under different types and degrees of stress, either through its own signaling or through constant signaling interactions with the nucleus and cytoplasm, a process known as the mitochondrial stress responses (MSRs). It is now believed that mitochondrial dysfunction is closely associated with a variety of muscle diseases caused by reduced levels of myogenic differentiation, suggesting the possibility that MSRs are involved in messaging during myogenic differentiation. Also, MSRs may be involved in myogenesis by promoting bioenergetic remodeling and assisting myoblast survival during myogenic differentiation. In this review, we will take MSRs as an entry point to explore its concrete regulatory mechanisms during myogenic differentiation, with a perspective to provide a theoretical basis for the treatment and repair of related muscle diseases.

## 1 Introduction

Skeletal muscle accounts for 30%–40% of healthy body weight and plays key roles in human voluntary movement, postural maintenance, respiration, and thermogenesis, the maintenance of which relies heavily on the regeneration and repair of skeletal muscle (Dumont et al., 2015; He et al., 2021). Skeletal muscle regeneration and repair is a complex process mediated by muscle satellite cells (MuSCs). MuSCs, located between the muscle membrane and the basal lamina of myofibers, are normally quiescent until they are activated by growth signals or injury stimuli. Upon activation, these cells will migrate and proliferate extensively to form mononuclear precursor cells, i.e., myoblasts. Subsequently, once the population of myoblasts has expanded, they exit the cell cycle and activate a differentiation programme to form multinucleated myotubes, which eventually fuse with existing myofibers to facilitate myofiber replenishment and repair. One of these key steps, the differentiation from myoblasts to myotubes, has been termed myogenic differentiation (Walsh and Perlman, 1997; Morgan and Partridge, 2003; Remels et al., 2010; Relaix and Zammit, 2012). This step is coordinated by a number of specific genes, such as myogenic regulatory factors (MRFs), including myogenic factor 5 (Myf5), myogenic regulatory factor 4 (MRF4), myoblast determination protein 1 (MyoD) and myogenin (MyoG), which direct progenitor cells to establish a myogenic lineage and activate the myogenic differentiation programme. MRFs also regulate the level of myosin heavy chain (MyHC), which determines the contractile properties of myofibers (Zammit, 2017). Reduced myogenic differentiation capacity has been reported to be associated with the progression of several muscle diseases, such as sarcopenia (Sun et al., 2022), skeletal muscle atrophy (Rahman and Quadrilatero, 2023) and Duchenne muscular dystrophy (Meyer et al., 2021).

Mitochondria are closely associated with myogenic differentiation due to the high energy requirements of skeletal muscle and the fact that mitochondria are the principal energy-supplying organelles in the cell (Wagatsuma and Sakuma, 2013). A striking feature of the process of myogenic differentiation is the change in the number, morphology and functional properties of mitochondria. Mitochondria in myoblasts are in an immature state, with low numbers, underdeveloped cristae and low levels of β-oxidation and overall respiration; whereas, when stimulated by differentiation factors, myoblasts must switch to a more oxidative phenotype to support a differentiation programme accompanied by metabolic reprogramming. As a result, mitochondria within differentiated myotubes appear more mature, with high numbers, enhanced levels of oxidative phosphorylation and increased ATP production (Vertel and Fischman, 1977; Barbieri et al., 2011; Rahman and Quadrilatero, 2021). To further elucidate the relationship between mitochondria and myogenic differentiation, several studies have used rotenone (complex I inhibitor), trifluoroacetone (complex II inhibitor) (Chabi et al., 2021), carbonylcyanide p-trifluoromethoxy-phenylhydrazone (FCCP) (mitochondrial uncoupler) (Wang et al., 2014), mitochondrial division inhibitor-1 (Mdivi-1) (dynamin-related protein 1 (Drp1) inhibitor) (Kim et al., 2013), ethidium bromide (EB) (mitochondrial DNA transcriptional inhibitor), rifampicin (mitochondrial RNA synthesis inhibitor) (Brunk and Yaffe, 1976), chloramphenicol (mitochondrial protein synthesis inhibitor) (Seyer et al., 2011), and UCF101 (High-Temperature Requirement Protein A2(HtrA2) inhibitor) (Sun et al., 2022) and others were used in differentiation model experiments, and it was found that affecting mitochondrial function from multiple aspects can have a negative impact on myogenic differentiation, indicating that mitochondrial diversity function controls the fate of myoblast differentiation.

When mitochondrial dysfunction occurs, mitochondria can continuously crosstalk with the nucleus and cytoplasm through signaling pathways originating from their own activation, activating a number of transcriptional, translational and post-translational programmes and pathways to execute multiple mitochondrial stress responses (MSRs). Short-term, mild stress can aid cellular homeostasis, whereas prolonged and severe stress can trigger apoptosis by causing an unresolved intracellular imbalance. Thus, the effect of MSRs is closely related to the duration and magnitude of the stress effects. (O'Malley et al., 2020; Picard and Shirihai, 2022). Furthermore, influenced by factors that initiate differentiation, skeletal muscle differentiation and formation itself is accompanied by significant levels of cellular stress changes such as caspase activation, increased reactive oxygen species (ROS), and upregulation of transcription of oxidative stress-related genes. (Jahnke et al., 2009; Baechler et al., 2019; Li et al., 2021; Gugliuzza and Crist, 2022), which confers the possibility that MSRs are involved in the transfer of information during myogenic differentiation. It is clear that MSRs play an important role in myogenic differentiation under both physiological and pathological conditions. Therefore, in this review, we describe multiple response pathways activated by mitochondrial stress, including integrated stress response (ISR), mitochondrial unfolded protein response (UPRmt), mitochondrial biogenesis, mitochondrial fusion and fission, mitophagy, apoptosis, as well as some of their known or potential effects on myogenic differentiation. With the aim of providing new perspectives and ideas to address related issues of muscle health and disease by exploring the link between MSRs and myogenic differentiation.

## 2 Selective transcriptional/translational effects of mitochondrial-nuclear interaction regulation on myogenic differentiation

### 2.1 ISR

In response to different stressful stimuli, eukaryotic cells activate a broader adaptive pathway known as the ISR. In mammals, four eukaryotic translation initiation factor 2alpha (eIF2α) kinases have now been identified, namely, general control non-derepressible 2 (GCN2), double-stranded RNA-dependent protein kinase (PKR), PKR-like ER kinase (PERK) and heme-regulated eIF2alpha kinase (HRI) (Donnelly et al., 2013). Stress activation of the four eIF2α kinases mentioned above phosphorylates the α-subunit of eIF2α at serine 51, and this phosphorylation blocks the formation of the ternary complex consisting of eIF2, GTP, and Met-tRNAi, which is a key step in 5′cap-dependent translation initiation, and thus can inhibit the overall translational initiation activity, leading to reduced protein synthesis (English et al., 2022). Next, ISR activation allows preferential expression of specific genes containing upstream open reading frames, such as the transcription factors activating transcription factor 4 (ATF4), C/EBP homologous protein (CHOP) and activating transcription factor 5 (ATF5), to ensure the subsequent generation of factors that aid in cellular recovery or, in some cases, initiate apoptosis (Eckl et al., 2021; Licari et al., 2021).

**FIGURE 1:**
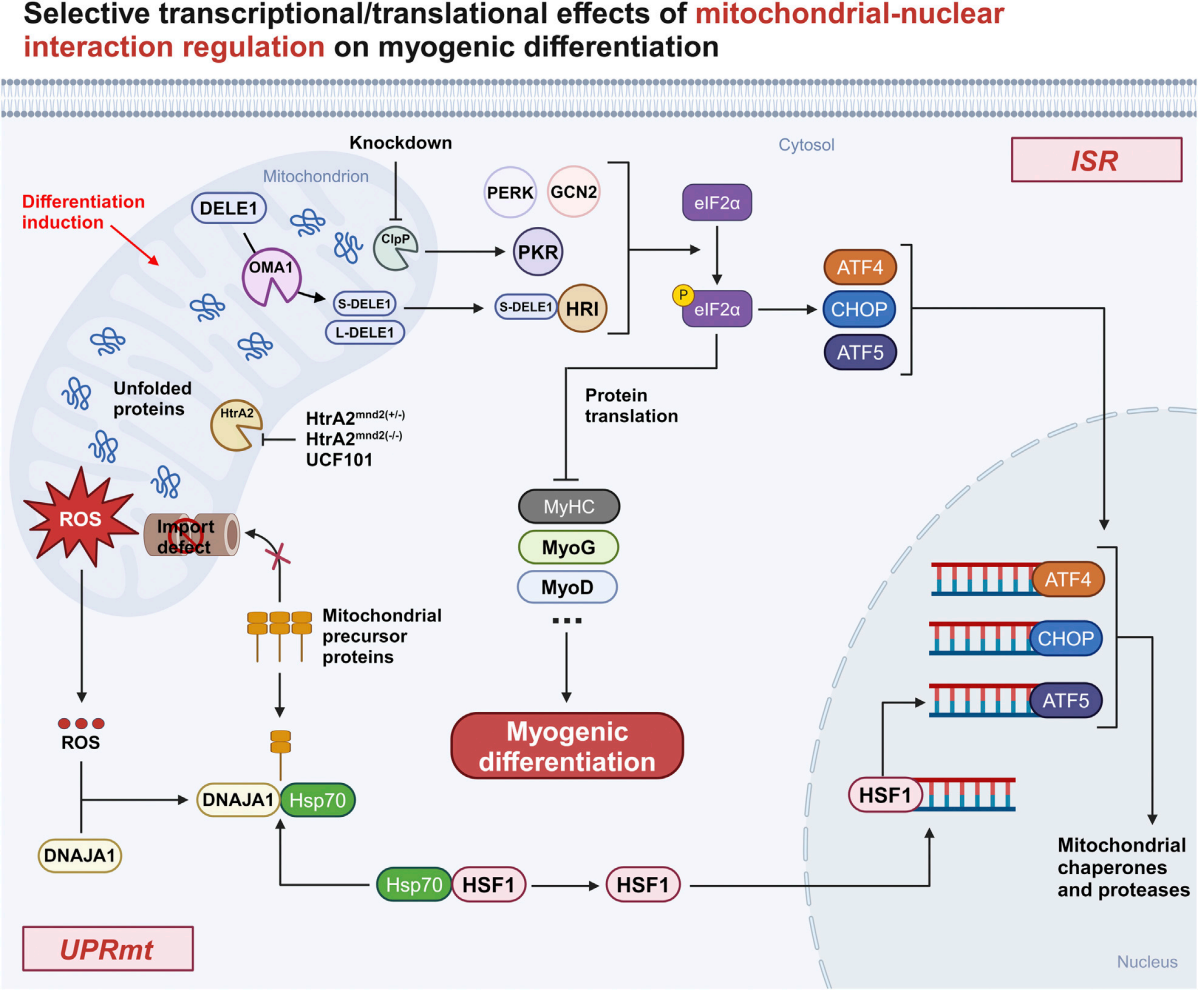
Selective transcriptional/translational effects of mitochondrial-nuclear interaction regulation on myogenic differentiation. Both knockdown ClpP and using UCF101 to inhibit HtrA2 or HtrA2 inactivation in Mnd2 mouse models show that activated mitochondrial-nuclear retrograde signaling inhibits myogenic differentiation by affecting the translation of myogenic genes. Abbreviation: ISR, integrated stress response; DELE1, DAP3 binding cell death enhancer-1; GCN2, general control non-derepressible 2; PKR, Double-stranded RNA-dependent protein kinase; PERK, PKR-like ER kinase; HRI, heme-regulated eIF2α kinase; eIf2α, eukaryotic translation initiation factor 2alpha; p-eIf2α, phosphorylation of eIf2α; ATF4, activating transcription factor 4; CHOP, C/EBP homologous protein; ATF5, activating transcription factor 5; UPRmt, mitochondrial unfolded protein response; DNAJA1, DnaJ homolog subfamily A member 1; HSP70, heat shock 70 protein; HSF1, heat shock factor 1; HtrA2, High-Temperature Requirement Protein A2; ClpP, caseinolytic protease P; MyHC, myosin heavy chain; MyoG, myogenin; MyoD, myoblast determination protein 1.

Of interest, a recent study by Guo X et al. (Guo et al., 2020) found that mitochondria can transmit stress signals into the cytoplasm via the OMA1-DAP3 binding cell death enhancer-1 (DELE1)-HRI pathway, which subsequently triggers ISR (Figure 1). Upon stress onset, DELE1 is cleaved by the inner mitochondrial membrane protease OMA1 and subsequently translocates to the cytoplasm to interact with HRI, which phosphorylates eIF2α, inhibits cytoplasmic protein translation, activates the transcription factors ATF4, CHOP, and ATF5, and initiates the expression of stress-related genes.

#### 2.1.1 ISR and myogenic differentiation

ATF4 is considered to be a key regulator of the ISR, and it is also involved in the regulation of myogenic differentiation. During myogenic differentiation, the transcription factor c-Myc is thought to promote myoblast proliferation and hinder myoblast differentiation by regulating the expression of its target genes, miRNAs and lincRNAs (Luo et al., 2019). Recently Memme JM et al. (Memme et al., 2023) found that ATF4 can affect myotube formation via c-Myc and MyoD and also control mitochondrial biogenesis via the PPAR-gamma coactivator-1alpha (PGC-1α). In addition, Sestrin2 (SESN2) is thought to be a downstream gene of ATF4 and is involved in mitochondrial stress-induced ISR (Garaeva et al., 2016). Piochi LF et al. (Piochi et al., 2021) proposed a theoretically possible role for SESN2 during myogenic differentiation: after the onset of differentiation, an increase in SESN2 expression, which induces the activation of AMP-activated protein kinase (AMPK) and the upregulation of nuclear factor erythroid 2-related factor 2 (NRF2) transcriptional levels, could enhance the efficiency of differentiation through three pathways:a) promotion of mitophagy b) generation of antioxidant responses c) increase in mitochondrial biogenesis. Meanwhile, another article (Brearley et al., 2019) noted that mRNA transcript levels of ISR-related genes, including SESN2 and ATF5, showed a similar trend with MyoG, reaching a maximum after about 48 h of induced differentiation and then starting to decline. It is suggested that SESN2 and ATF5 may coordinate MyoG to play some roles in the differentiation of the mouse myoblast cell line C2C12, but experiments are still needed to further verify this.

CHOP is an important transcription factor regulating the ISR, and Alter J et al. (Alter and Bengal, 2011) demonstrated that CHOP can inhibit MyoD transcription to delay myoblast differentiation. Normally, CHOP expression is downregulated early in the differentiation of C2C12 cells. Upon knockdown of CHOP, the fusion index and differentiation rate of C2C12 cells were significantly increased. In contrast, overexpression of CHOP significantly delayed C2C12 cell differentiation, and the resulting result was caused by CHOP inhibition of MyoD. This suggests that downregulation of CHOP expression is required for the activation of the myogenic differentiation programme in C2C12 myoblasts.

Although no direct evidence for the involvement of ATF5 in myogenic differentiation was found, the differentiation effects of ATF5 for tissues such as bone, liver, brain and adipose were well characterised (Sears and Angelastro, 2017). This suggests that ATF5 has more potential roles in developmental and normal physiological processes, and may provide new research directions for exploring the regulatory mechanisms of myoblast proliferation and differentiation.

The effect of the OMA1-mediated DELE1-HRI axis on myogenic differentiation has not been reported in detail. Whether the OMA1-mediated DELE1-HRI axis is then a necessary pathway for the activation of the ISR-associated transcription factors ATF4, CHOP, and ATF5 involved in myogenic differentiation is worth exploring subsequently. Although, a direct effect of this signaling axis on myogenic differentiation was not seen, it was described in a study of mitochondrial myopathy. CHCHD10, a nuclear gene-encoded mitochondrial protein, is mainly situated in the mitochondrial intermembrane space (IMS) and, in conjunction with its homologue CHCHD2, has a vital function in preserving mitochondrial inner membrane integrity and electron transport chain (ETC) function (Jiang et al., 2022). Mutations in the CHCHD10 G58R gene can lead to mitochondrial myopathy. According to this study (Shammas et al., 2022), OMA1 is activated in response to misfolding of the CHCHD10 G58R protein. This activation triggers the ISR through the OMA1-DELE1-HRI signaling pathway, ultimately providing a protective effect that prolongs the survival of newborn infants with CHCHD10 G58R gene mutations. Given the correlation between mitochondrial myopathy and myogenic differentiation, this suggests that the OMA1-mediated DELE1-HRI axis may have an effect on myogenic differentiation.

### 2.2 UPRmt

UPRmt was discovered by Hoogenraad’s laboratory as early as 1996 (Martinus et al., 1996). As an adaptive intracellular stress mechanism, it primarily responds to stress signals by promoting the transcription of genes for nuclear DNA-encoded mitochondrial chaperone proteins and proteases in order to maintain mitochondrial proteostasis. Among them, mitochondrial chaperone proteins, which enable newly synthesised proteins to fold correctly and help misfolded proteins to restore their normal conformation, mainly include heat shock protein 70 (HSP70), heat shock protein 60 (HSP60), heat shock protein 10 (HSP10), and others. Whereas proteases are capable of degrading damaged proteins, the main ones include caseinolytic protease P (ClpP), YME1 Like 1 ATPase (YME1L1) and Lon protease 1 (LonP1) (Zhu et al., 2021).

How does UPRmt transmit information about mitochondrial misfolding stress (MMS) to the nucleus to exert its effects? A recent study by Sutandy FXR et al. (Sutandy et al., 2023) gave the explanation that MMS can lead to the accumulation of mitochondrial protein precursors in the cytosol (c-mtProt), while at the same time, MMS can contribute to the release of mitochondrial reactive oxygen species (mtROS) into the cytosol and oxidation of the HSP40 family protein DnaJ homolog subfamily A member 1 (DNAJA1). Subsequently, binding of HSP70 to oxidised DNAJA1 resulted in enhanced recruitment of HSP70 to c-mtProt. This process leads to a reduction in the binding of HSP70 to the conventional chaperone heat shock factor 1 (HSF1), and the released HSF1 translocates to the nucleus to activate ATF5 transcription, thereby triggering the anti-stress mechanism (Figure 1).

#### 2.2.1 UPRmt and myogenic differentiation

ClpP is an important protease within the mitochondrial matrix involved in the initiation of UPRmt. ClpP expression is higher in skeletal muscle compared to other tissues (Bross et al., 1995). In C2C12 cells after ClpP knockdown (KD), Drp1 was upregulated, leading to altered mitochondrial morphology, while the ETC subunit protein expression level was reduced, attenuating the activity of the ETC complex and thus affecting mitochondrial respiration, suggesting that ClpP is essential for maintaining mitochondrial function in myoblasts (Deepa et al., 2016). In addition, treatment of ClpP KD C2C12 cells using doxycycline (UPRmt inducer) showed no upregulation of the UPRmt-associated protein Hsp60 in both the dosed and undosed groups, suggesting that ClpP is one of the major pathways for mitochondrial stress-induced activation of UPRmt. Moreover, in the experimental group of ClpP KD C2C12 cells, enhanced expression levels of PKR and phosphorylation of eIf2α (p-eIf2α) were also found, whereas the expression of myogenic differentiation-associated proteins, such as MyHC and MyoG, was not upregulated, suggesting that myogenic differentiation is significantly impaired when protein translation is inhibited (Deepa et al., 2016). The aforementioned findings indicate that ClpP has an impact on myogenic differentiation via UPRmt and also plays a role in regulating myogenic differentiation through ISR. This highlights the strong correlation between UPRmt and ISR.

HtrA2 is a protease located in the mitochondrial IMS that is involved in UPRmt to maintain mitochondrial homeostasis (Riar et al., 2017). Previously published results from our team (Sun et al., 2022) showed that inhibition of HtrA2 enzyme activity using UCF101 resulted in blocked differentiation of C2C12 myoblasts. Considering that the loss of HtrA2 enzyme activity-induced proteostasis imbalance and mitochondrial IMS stress are the main triggers of UPRmt, we hypothesised that UPRmt could be associated with impaired differentiation due to HtrA2 enzyme activity inhibition. To this end, C2C12 cells in the differentiated state were treated with UCF101, and Western blotting results showed upregulation of the expression of the UPRmt-related protein CHOP, the HSP family of molecular chaperones, and the LonP1 protein. This suggests that loss of HtrA2 enzyme activity activates UPRmt to inhibit the translation of myogenic differentiation-related proteins, resulting in blocked myogenic differentiation.

The effect of the mtROS/c-mtProt-driven DNAJA1-HSF1 axis on myoblast differentiation has not been reported in detail. However, relevant signaling molecules targeting this axis have been described. A study (Kim et al., 2018) indicated that mtROS rapidly increases during differentiation and that mtROS can activate the phosphatidylinositol three kinase (PI3K)/AKT/mammalian target of rapamycin (mTOR) signaling pathway by inducing tensin homolog deleted on chromosome 10 (PTEN) oxidation. After activation, mTORC1 promotes phosphorylation at uncoordinated-51 like kinase 1 (ULK1) S317 and upregulates the expression of Atg protein, leading to autophagy and participating in the myogenic differentiation process. In addition, several studies have shown that heat shock proteins (HSPs) are involved in myogenic differentiation (Table 1). While muscle repair is significantly diminished in aged mice undergoing severe injury, aged mice overexpressing HSP70 reversed this outcome, which may be related to the promotion of myoblast fusion by HSP70 (McArdle et al., 2006; Thakur et al., 2020). HSPs are transcriptionally regulated by two members of the heat shock transcription factor family, HSF1 and heat shock factor 2 (HSF2), with HSF1 functioning primarily by responding to a variety of stressful environments, while HSF2 functions under non-stressful conditions such as differentiation and development (Santopolo et al., 2021). It has been shown (McArdle et al., 2006) that during myogenic differentiation, HSF1 levels increase significantly in the late stages, whereas HSF2 levels increase rapidly in the early stages and then return to normal levels. It is suggested that the early development and recombination of skeletal muscle cells is regulated by HSF2, and the ability of cells to respond to acute stress depends mainly on HSF1 and the UPRmt regulated by it.

**TABLE 1:** Heat shock proteins (HSPs) are involved in myogenic differentiation.

Protein	Subcellular localization	Expression changes during myogenic differentiation	Participating processe/function	PMID
Hsp25	Cytoplasm	↓	Involved in myofiber assembly	31098840
Hsp40	Cytoplasm, Nucleus	↓	Control of myoblast cycle exit	31098840
Hsp60	Translocation from perinuclear to cytoplasm	↓	Inhibition of myotubes apoptosis	31098840, 28274921
Hsp70	Cytoplasm, Nucleus	↑	Driving myoblast fusion	32605905, 30275345
Hsp90	Cytoplasm	—	Supporting myoblast survival	31098840, 21739150

In conclusion, both ISR and UPRmt are mitochondria-nuclear interactive regulatory responses, which can activate the same signaling molecules to exercise different signaling pathways (Roca-Portoles and Tait, 2021). Some literature indicates that in mammals, ISR is a prerequisite for the induction of UPRmt, as the expression of the three basic leucine zip (bZIP) transcription factors ATF4, CHOP, and ATF5 involved in UPRmt requires the activation of ISR (Rainbolt et al., 2013; Melber and Haynes, 2018; Anderson and Haynes, 2020). A typical example is arsenite-induced MSRs (Rainbolt et al., 2013), in which arsenite first activates the ISR and the transient phosphorylation of eIF2α leads to a reduction in Tim17A synthesis, which reduces the import efficiency of mitochondrial proteins and induces the expression of UPRmt-related genes. It has also been noted in the literature (Khan et al., 2017) that UPRmt is part of ISRmt. This interactive regulation at the level of front and back, whole and part, reflects the close connection between the two. Even though both are “saviours” of the cell against stress, myogenic differentiation is affected during differentiation, whether they are over-activated or inhibited (Figure 1). This is because the general translational decline caused by overactivation affects the expression of differentiation-related proteins, and overrepression weakens the ability of cells to withstand stress during differentiation. It is thus clear that the interactive effects between such signaling pathways induced by the stress response are extremely complex, and the mechanisms of their regulation on myogenic differentiation still need to be further explored.

## 3 The role of mitochondrial dynamics in remodeling the mitochondrial network during myogenic differentiation

### 3.1 Mitochondrial biogenesis

Mitochondrial biogenesis is the process of generating new mitochondria from existing mitochondria and is regulated by both the mitochondrial and nuclear genomes (Scarpulla, 2008). The peroxisome proliferator-activated receptor-gamma coactivator-1 (PGC-1) family includes three members, PGC-1α, PGC-1β and PGC related coactivator, of which PGC-1α is considered to be a master regulator of mitochondrial biogenesis, binding to and enhancing the activity of transcription factors such as NRF1/2, ERR alpha, *etc.*, causing NRF1/2 to activate the expression of TFAM, TFB1M, TFB2M and a number of mitochondrial matrix proteins to drive the replication and transcription of mtDNA and its translation into proteins and ultimately assembly into new mitochondria (Scarpulla, 2008; Fernandez-Marcos and Auwerx, 2011).

#### 3.1.1 Mitochondrial biogenesis and myogenic differentiation

The process of myogenic differentiation is accompanied by increased levels of mitochondrial biogenesis, resulting in an increase in the number and activity of mitochondria, enabling myoblasts to adapt to stressful conditions and increase cell viability (Fortini et al., 2016; Piochi et al., 2021). As a transcriptional co-activator, the role of PGC-1α in myogenic differentiation has been elucidated. PGC-1α serves a dual role in both triggering mitochondrial biogenesis to increase mitochondrial mass and buffering the expression of genes involved in mitophagy mediated by ROS/FOXO1 (Baldelli et al., 2014). This not only indicates that PGC-1α is an important integrating molecule for mitochondrial biogenesis and mitophagy, but also suggests that there is a close collaboration between mitochondrial biogenesis and mitophagy during myogenic differentiation. A study (Sin et al., 2016) showed that downregulating PGC-1α in C2C12 myoblasts increases ROS generation, causes mitochondrial damage, and leads to poor differentiation. In contrast, in vivo studies in mice, upregulation of PGC-1α expression alters MRFs and exhibits a positive protective effect on skeletal muscle regeneration by promoting myogenic differentiation (Washington et al., 2022). In addition, some important transcription factors, such as NRF1/2 and TFAM, have been found to show a trend of transcriptional upregulation during differentiation (Kraft et al., 2006; Remels et al., 2010), and NRF1 has been shown to mediate PGC-1β-induced mitochondrial biogenesis and activation of cellular respiration in conjunction with ERR alpha (Shao et al., 2010). So far, a number of studies have shown that activation of mitochondrial biogenesis-related signaling pathways can promote myogenic differentiation (Li et al., 2007; Barbieri et al., 2016; Lee and Choi, 2018; Niu et al., 2021; You et al., 2023). Thus, mitochondrial biogenesis plays an important role in myogenic differentiation.

### 3.2 Mitochondrial fusion and fission

Dynamic changes in mitochondria provide a wide range of benefits to mitochondria, including efficient transport, increased homogeneity of the mitochondrial population, and efficient oxidative phosphorylation. These benefits are achieved through mitochondrial fusion and fission, which control mitochondrial number, morphology, equitable inheritance of mitochondria, material exchange and maintenance of a high quality mitochondrial genome, as well as degradation of isolated bioenergetically inefficient mitochondria by mitophagy (Chan, 2020). Relevant molecules that mediate mitochondrial fusion and fission include mitofusin-1 (MFN1), mitofusin-2 (MFN2), optic atrophy 1 (OPA1), Drp1 and mitochondrial fission 1 (Fis1), signaling molecules that endow mitochondria with the ability to sense and respond to a variety of environmental conditions (Humphries et al., 2020).

#### 3.2.1 Mitochondrial fusion and fission and myogenic differentiation

**FIGURE 2:**
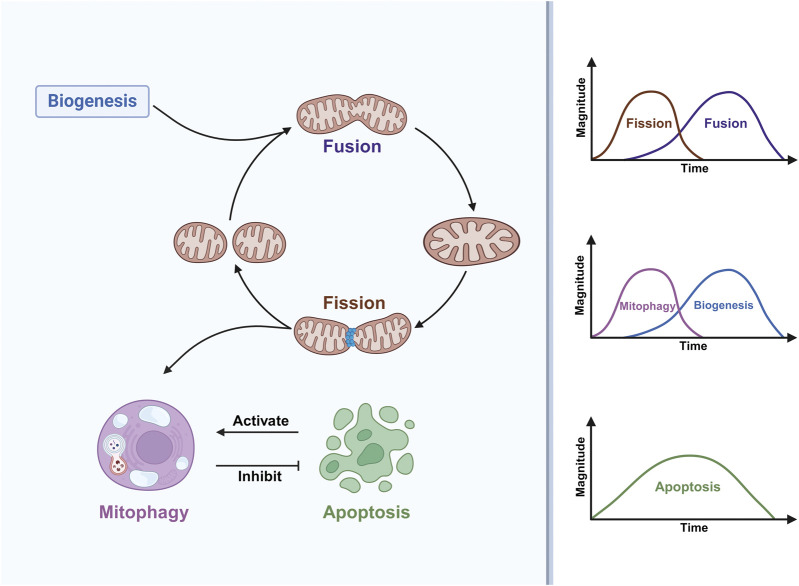
Mitochondrial dynamics are involved in myogenic differentiation. Mitochondrial fission is a prerequisite for the occurrence of mitophagy, which inhibits apoptosis, and apoptosis in turn activates mitophagy. During myogenic differentiation, mitochondrial fission and mitophagy occur early, mitochondrial fusion and mitochondrial biogenesis occur in the middle and late stages, while apoptosis persists throughout differentiation, with a high level of apoptosis in the middle of differentiation.

There is a link between mitochondrial dynamics and myogenic differentiation (Figure 2). In the early stages of muscle differentiation, mitochondrial fission is activated while fusion is inhibited, resulting in a loose mitochondrial network; in the later stages, the opposite occurs, resulting in a tight mitochondrial network (Huang et al., 2020). This fully reflects the adaptive response of mitochondrial fusion and fission activity to environmental changes during myogenic differentiation. It was recently shown (Luo et al., 2021) that the expression of MFN2 proteins involved in mitochondrial fusion is upregulated during myogenic differentiation, and that myoblasts isolated from MFN2-null mice exhibit abnormal mitochondrial respiration with elevated ROS. However, interestingly, these alterations do not negatively affect the proliferation and differentiation of myoblasts, which may be due to a potential compensatory function of MFN1 that prevents these alterations from affecting muscle development and regeneration (Luo et al., 2021). In conjunction with some existing work a) inhibition of dihydroorotate dehydrogenase (DHODH) activity increases MFN2 expression and promotes mitochondrial fusion via the pyrimidine *de novo* synthesis pathway (Miret-Casals et al., 2018) b) knockdown of MFN2 limits the synthesis and consumption of aspartate, a raw material for the pyrimidines *de novo* synthesis (Yao et al., 2019) c) DHODH is localised in the inner mitochondrial membrane and is linked to the respiratory chain via the coenzyme Q pool and is involved in oxidative phosphorylation (Boukalova et al., 2020) d) DHODH in skeletal muscle mitochondria can produce H_2_O_2_ directly or indirectly (Hey-Mogensen et al., 2014). This suggests that there may be a feedback mechanism for MFN2 knockout to affect DHODH activity through the pyrimidine *de novo* synthesis pathway. If so, can it be argued that the abnormal mitochondrial respiration and elevated ROS exhibited in MFN2-null cells may be caused by DHODH affecting the mitochondrial respiratory chain? Further work is needed to verify this. This further suggests the potential involvement of DHODH in myogenic differentiation through the regulation of MFN2-mediated mitochondrial fusion. In addition to MFN2, another key molecule of mitochondrial fusion, OPA1, has also been reported to have an effect on myogenic differentiation, and cells under oxidative stress induced by the application of tert-butylhydroperoxide (t-BHP) or FCCP treatments to C2C12 cells can inhibit myogenic differentiation by cleaving OPA1 to reduce MyHC expression (Wang et al., 2014).

The effect of mitochondrial fission activity on myogenic differentiation has been described. One study (Kim et al., 2013) noted that inhibition of Drp1 activity using Mdivi-1 impairs myogenic differentiation. Interestingly, another study (De Palma et al., 2010) pointed out that nitric oxide inhibition of Drp1-mediated mitochondrial fission is an essential step in the process of early myogenic differentiation, which seems to contradict the previous findings. Further studies are needed to fully explain this discrepancy. In addition, a study on muscle regeneration after frostbite (Wagatsuma et al., 2011) found that Fis1 expression was upregulated early in muscle regeneration, along with its concomitant upregulation of the myogenic differentiation-associated proteins MyoD and MyoG. This suggests that mitochondrial fission may not only regulate myogenic differentiation by affecting mitochondrial dynamics and morphological alterations, but also that mitochondrial fission-associated proteins may be involved as signaling molecules in regulating the expression of factors associated with myogenic differentiation. These findings re-emphasise that tight regulation of the activity of mitochondrial fusion and fission proteins and, more importantly, the maintenance of a dynamic balance between mitochondrial morphology and function are essential for the initiation and progression of myogenic differentiation.

### 3.3 Mitophagy

The concept of mitophagy was first formalised by Lemasters JJ in 2005 (Lemasters, 2005). When stress damage is severe, mitochondria signal that they can remove damaged mitochondria by mitophagy on their own, thus preventing further damage to the mitochondrial network caused by stress. Mitophagy is a degradation process that begins with the engulfment of mitochondria into a double-membrane structure known as an autophagosome, and finally completes the degradation after fusion with lysosomes (Picca et al., 2021). In general, mitophagy can be divided into the sequestosome-like receptor (SLR)-dependent mitophagy pathways mediated by PINK1 and the E3 ubiquitin ligase PRKN, and SLR-independent mitophagy pathway**s** mediated by autophagy receptors, specific classes of lipids, and partial E3 ubiquitin ligases (Sun et al., 2021; Ganley and Simonsen, 2022).

#### 3.3.1 Mitophagy and myogenic differentiation

McMillan EM et al. (McMillan and Quadrilatero, 2014) demonstrated for the first time that myogenic differentiation requires the involvement of autophagy, which prevents apoptosis generated during myogenic differentiation. Afterwards, some workers directed their research specifically to mitophagy to explore the link between mitophagy and myogenic differentiation. Sin J et al. (Sin et al., 2016) proposed that the shift in energy metabolism from glycolysis to oxidative phosphorylation during the differentiation of myoblasts to myotubes requires the involvement of mitophagy. Notably, Brown HMG et al. found that mitophagy is a transient phenomenon that is specifically upregulated only in the early stages of differentiation (Figure 2), whereas the overall cellular autophagic flux persists throughout the differentiation phase (Brown et al., 2021). Mitochondrial network remodeling is an important feature of myogenesis (Rahman and Quadrilatero, 2021). One study (Li et al., 2022) claimed that tetrandrine can inhibit myogenic differentiation by blocking autophagic flux leading to impaired mitochondrial network remodeling, which shows that autophagy-mediated mitochondrial network remodeling can also have an effect on myogenic differentiation. The impact of mitophagy on the entire myogenic differentiation event is profound, specifically regulating multiple aspects of mitochondrial network remodeling, oxidative stress, ER-associated stress, mitochondria-mediated apoptotic signaling that occur during differentiation (Baechler et al., 2019; Jiang et al., 2021).

(1) Impact of SLR-dependent mitophagy pathways on myogenic differentiation

PINK1, an important participant in mitophagy (Lazarou et al., 2015), has been shown to affect myoblast differentiation, and interfering with PINK1 using siRNAs in C2C12 cells leads to a decrease in the transcriptional level of MyoG (Baldelli et al., 2014). Similarly, PRKN, another important player in mitophagy, has now been shown to be recruited into mitochondria during early differentiation of C2C12 (Esteca et al., 2020), which supports the conclusion that mitophagy is involved in early myogenic differentiation as suggested by Sin J et al. (Sin et al., 2016). Importantly, PRKN deficiency results in myoblasts remaining in a proliferative state, delayed differentiation and even a reduction in myofiber area (Peker et al., 2018; Esteca et al., 2020); in contrast, overexpression of PRKN has been shown to increase muscle mass and strength in aged mice (Leduc-Gaudet et al., 2019). This suggests that PRKN is involved in the regulation of skeletal muscle growth and development. Moreover, Huang S et al. (Huang et al., 2020) found that LonP1 regulates mitochondrial network remodeling through the PINK1/PRKN-mediated mitophagy pathway to promote myogenic differentiation. This suggests that the regulatory role of Lonp1 on myogenic differentiation is not only limited to its role in UPRmt, but its role in mitophagy is also involved. It indicates that Lonp1 may be an integrated molecule of different MSRs during myogenic differentiation.

(2) Impact of SLR-independent mitophagy pathways on myogenic differentiation

Baechler BL et al. (Baechler et al., 2019) used CRISPR-Cas9 technology to construct a Bcl-2/adenovirus E1B 19 kDa interacting protein 3 (BNIP3)^−/−^ C2C12 cell model, and the experimental results showed that knockout of BNIP3 impaired myogenic differentiation, which was associated with mitophagy defects. And supplementation of Insulin-like growth factor 1 could attenuate the impaired myogenic differentiation caused by defective mitophagy (Guan et al., 2022). Meanwhile, knockout of BNIP3 also caused increased caspase-3 and caspase-9 activities and DNA fragmentation, which could lead to apoptosis of myoblasts. Another mitophagy receptor, FUN14 domain containing 1 (FUNDC1), has not been found to have a direct correlation with myogenic differentiation but has been shown to be closely related to skeletal muscle mitochondrial function, and FUNDC1 deficiency leads to defects in mitophagy mediated by microtubule-associated protein light chain 3 (LC3), causing impaired skeletal muscle mitochondrial function (Fu et al., 2018). Of interest, FUNDC1 not only mediates mitophagy in skeletal muscle cells involved in the differentiation process, but also plays a role in the differentiation process of human cardiac progenitor cells (Lampert et al., 2019).

### 3.4 Apoptosis

Apoptosis is a conserved physiological mechanism responsible for eliminating dysfunctional, damaged or unwanted cells in order to precisely control cell numbers. The signaling pathway that initiates endogenous apoptosis is triggered by mitochondria, and key to the regulation and execution of this event is the B-cell lymphoma/leukaemia-2 (Bcl-2) family of proteins (Rosa et al., 2022). When confronted with stress stimuli such as DNA damage, cellular hypoxia, and cellular growth factor deficiency, cells can activate BH3-only proteins, which subsequently inhibit the Bcl-2 family of anti-apoptotic proteins, activate free Bax and Bak to form oligomers at the outer mitochondrial membrane, resulting in the outer mitochondrial membrane permeabilization (MOMP), and release cytochrome c (Cytc) into the cytoplasm to bind with apoptotic protease activator factor 1 (Apaf-1) to form apoptosomes, which activate the cascade of responses mediated by the caspase family proteins to induce apoptosis. MOMP also leads to the release of second mitochondria-derived activator of caspases (SMAC) and HtrA2 proteins, both of which inhibit the anti-apoptotic effects of the X-linked inhibitor of apoptosis protein (XIAP), thereby promoting caspase-independent apoptosis (Bock and Tait, 2020; Lee et al., 2022).

#### 3.4.1 Apoptosis and myogenic differentiation

Apoptosis-related events (e.g., cytoskeletal reorganization (Qu et al., 1997), phosphatidylserine receptor expression (Hochreiter-Hufford et al., 2013)) are necessary for myogenic differentiation. However, either excessive or absent apoptosis affects myotube formation (Fernando et al., 2002), suggesting that the level of apoptosis must be tightly regulated during myogenic differentiation.

(1) Impact of Bcl-2 family-related members on myogenic differentiation

Rahman FA et al. (Rahman and Quadrilatero, 2023) pointed out that in the early stage of myogenic differentiation, the local anti-apoptotic protein Bcl-2 levels in mitochondria rapidly increase, which may be related to the increased resistance of myoblasts to stressors produced during the initial differentiation process. The mitochondrial Bcl-2 inhibitor of transcription 1 (Bit1) was shown to be an essential mediator of muscle differentiation. Bit1-null mice exhibit a smaller muscle cross-sectional area, and primary myoblasts isolated from this model mice, with barely detectable Bcl-2, instead show higher caspase-3 activity and premature onset of myogenic differentiation (Griffiths et al., 2015). In addition to Bit1, Bcl-2 levels are regulated by mitogen-activated protein kinase (MAPK) signaling, and its effect on Bcl-2 is associated with skeletal muscle regeneration. For example, upregulation of Bcl-2 expression induced by MAP kinase phosphatase 5 (MKP-5) inhibition promotes the survival of regenerating myofibers (Min et al., 2017; Rahman and Quadrilatero, 2023). Another Bcl-2 family protein, B-Cell Lymphoma-extra-large (Bcl-xL), whose overexpression has the same effect on muscle differentiation as knockdown of caspase-9, inhibits myoblast fusion (Murray et al., 2008). In addition, the pro-apoptotic BH3-only protein BCL2 binding component 3 (BBC3/PUMA) appears transiently elevated 1 day after differentiation of C2C12 cells, with a concomitant increase in P53 (Rahman and Quadrilatero, 2023). Another study (Harford et al., 2017) noted that both MyoD and P53 bind to the BBC3 promoter when stimulated by differentiation conditions, and silencing MyoD prevents binding from occurring. These data demonstrate a complex link between the three and support an important role for Bcl-2 family-related members regulating mitochondrial apoptotic signaling during myogenic differentiation for effective differentiation and myofiber regeneration.

(2) Impact of the caspase family on myogenic differentiation

Early work found that activation of caspase-3 was required for myoblast differentiation. Primary myoblasts isolated from caspase-3 null mice show normal proliferative capacity, however, after induction of differentiation, these cells continue to express cytokinin D1 (CCND1), resulting in an inability to exit the cell cycle and impaired differentiation (Fernando et al., 2002). Further subsequent studies demonstrated that caspase-3 activation during differentiation is caused by caspase-9 and found that knockdown of caspase-9 significantly affected caspase-3 activation and inhibited myoblast fusion (Murray et al., 2008). Cytc release is a key step in the activation of caspase-9, and indeed, myoblasts show Cytc release shortly after differentiation, accompanied by elevated caspase-9 activity (Rahman and Quadrilatero, 2023). C2C12 myoblasts after knockdown of Cytc using siRNA resulted in reduced apoptotic body formation, preventing caspase-9 and subsequent caspase-3 activation to reduce myotube formation (Dehkordi et al., 2020). Finally, caspase-2 and p21 have been found to be activated during the early stages of myogenic differentiation, which may underlie the proper cell cycle exit of myoblasts and the subsequent occurrence of differentiation (Boonstra et al., 2018).

In summary, myogenic differentiation is a highly dynamic and tightly coordinated process, with mitochondrial dynamic processes such as mitochondrial biogenesis, mitochondrial fission, fusion, mitophagy, and apoptosis running through the entire process of myogenic differentiation (Figure 2), which regulates the mass, structure, function and distribution of mitochondria. Myogenic differentiation coupled with metabolic reprogramming ultimately leads to an increase in mitochondrial mass and OXPHOS to support newly formed myotubes, which requires replacement of the original mitochondria of myoblasts (Rahman and Quadrilatero, 2021). Thus, mitochondrial fission and subsequent activation of mitophagy are critical processes in the early differentiation of myoblasts. Interestingly apoptosis activates mitophagy, which in turn inhibits apoptosis, thus maintaining the stability of myogenic differentiation (McMillan and Quadrilatero, 2014; Baechler et al., 2019; Jiang et al., 2021). After pre-existing mitochondria are removed, mitochondrial biogenesis and mitochondrial fusion drive the formation of a dense network of mitochondria to complement and sustain cellular energy metabolism (Sin et al., 2016; Rahman and Quadrilatero, 2021). All these results suggest that the individual stress responses during myogenic differentiation are not separate entities but an intricate network. In addition, it is worth noting that the mitochondrial dynamics processes associated with the above are regulated by Ca^2+^ signaling. For example, Ca^2+^ stimulates mitochondrial biogenesis (Yeh et al., 2005). Ca^2+^ can regulate mitochondrial fusion and fission homeostasis by mediating Drp1 phosphorylation or dephosphorylation (Cribbs and Strack, 2007; Cereghetti et al., 2008). Mitochondrial permeability transition pore (mPTP) opening is a crucial event in the induction of mitophagy and apoptosis, and Ca^2+^ overload is one of the key factors in inducing mPTP opening (Rimessi et al., 2013). Interestingly, the current study also found that Ca^2+^ and its associated signaling molecules are strongly associated with myogenic differentiation. Earlier studies have shown that myogenic differentiation is accompanied by transmembrane inward flow of extracellular Ca^2+^ and that lowering extracellular Ca^2+^ concentrations inhibits myoblast fusion (Adamo et al., 1976; Naro et al., 2003). Sun W et al. (Sun et al., 2017) proposed that intracellular Ca^2+^ may promote myogenic differentiation via the MLCK-MLC-myosin-actin pathway, a signaling pathway that mediates cytoskeletal dynamics. Meanwhile, some important molecules involved in the regulation of Ca^2+^ signaling, such as calcineurin, may be involved in the early process of myogenic differentiation by regulating nuclear factor of activated T cells 3(NFATc3) to promote MyHC expression (Delling et al., 2000); MyoG expression requires the activation of calmodulin-dependent protein kinase II (CaMII) (Terruzzi et al., 2017); S100 calcium binding protein B (S100B) is a chiral EF Ca^2+^-binding protein that is downregulated in the early stages of myogenic differentiation as an inhibitor of myogenic differentiation, whereas in the late stages S100B expression is upregulated and reduces apoptosis through activation of NF-κB (Tubaro et al., 2011). It remains to be elucidated whether Ca^2+^ can be involved in the regulation of myogenic differentiation by influencing mitochondrial dynamics.

## 4 Conclusion and future perspectives

During myogenesis, myoblasts go through several important stages: first, rapid proliferation, followed by exit from the cell cycle, then myogenic differentiation and finally cell fusion to form multinucleated myofibers. Here, we highlight the impact of stress responses related to mitochondrial-nuclear interaction regulation signals and mitochondrial dynamics on myogenic differentiation (Table 2), and we argue that moderate stress signals are required to support myogenic differentiation. At the same time we can see that in the last decades there has been a rapid growth in the understanding of mitochondrial biogenesis, mitochondrial fusion and fission, mitophagy and apoptosis related to mitochondrial dynamics in myogenic differentiation, whereas there is still a lack of understanding of ISR, UPRmt related to the regulation of mitochondrial-nuclear interactions. For example, rapid dephosphorylation of eIF2α occurs early in differentiation and the ISR is inhibited (Zismanov et al., 2016), whereas ATF5 expression is elevated during the same period, suggesting to some extent that the UPRmt is activated (Fiorese et al., 2016), and whether this phenomenon could indicate that the activation of the UPRmt is not dependent on the ISR under certain conditions, and that this link has been pointed out in several previous studies (Münch and Harper, 2016; Forsström et al., 2019; Sutandy et al., 2023). In addition, as mentioned previously, ATF5 has a potential role in development and normal physiology, suggesting that the way in which ATF5 is activated may not be limited to stress onset. Therefore, these outstanding issues still require our attention. Further, a common feature of MSRs and myogenic differentiation is that both require complex signaling cascades, suggesting that we also need to consider the role of organelles other than mitochondria in myogenic differentiation. Finally, it has been reported (Zismanov et al., 2016) that phosphorylation of eIF2α is a translational regulatory mechanism that regulates the quiescence and self-renewal of MuSCs, suggesting that myogenic differentiation is only one stage in the process of skeletal muscle regeneration and repair, and that the roles played by MSRs in the other stages of myogenesis need to be considered. These research gaps can be explored and analysed in depth in order to gain a comprehensive understanding of this knowledge and to provide theoretical support for the subsequent treatment of related muscle disorders.

**TABLE 2:** Mechanisms of regulation of myogenic differentiation by MSRs-related molecules.

Type	Molecule	Mechanism	PMID
ISR	ATF4	Influencing on myotube formation via c-Myc and MyoD	37273238
	CHOP	Regulation of MyoD gene transcription and protein expression	22242125
UPRmt	ClpP	Influencing mitochondrial morphology and respiration in myoblasts	26721594
		Activation of ISR to affect MyHC, MyoG protein translation	
	HtrA2	Activation of UPRmt to affect MyoG, Myosin protein translation	36233059
	LonP1	Regulation of mitochondrial network remodeling through the PINK1/PRKN-mediated mitophagy pathway	32936696
Mitochondrial biogenesis	PGC-1α	Triggering mitochondrial biogenesis	25375380
		Buffering the expression of genes involved in ROS/FOXO1-mediated mitophagy	
	NRF1	Collaborating with ERR alpha to mediate PGC-1β induced mitochondrial biogenesis and activation of cellular respiration	20561910
Mitochondrial fusion and fission	MFN2	Regulation of mitochondrial fusion in late myogenic differentiation	33749882
	OPA1	Influencing MyHC protein translation	25393477
	Drp1	Regulation of mitochondrial fission in early myogenic differentiation	23904108
Mitophagy	PINK1	Mediating mitophagy and affects MyoG protein transcription	25375380
	PRKN	Mediating mitophagy in skeletal muscle regeneration	33126429
	BNIP3	Influencing caspase-3 and caspase-9 activity and regulates apoptosis	30859901
Apoptosis	Bcl-2	Resistance to stress in the early stages of myogenic differentiation	35241367
	Bcl-xL	Influencing myoblast fusion	18957517
	Caspase-3	Influencing cell cycle progression	12177420
	Caspase-9	Influencing myoblast fusion	18957517
	Cytc	Influencing caspase-3 and caspase-9 activity	32366831
	Caspase-2	Regulating p21 induction at early stages of myogenic differentiation	28765049

Experimental animal studies have shown that some drugs can improve the disease phenotype by activating MSRs with significant efficacy. For example, nicotinamide riboside (NR), as a precursor of NAD+, can be involved in maintaining mitochondrial proteostasis in amyotrophic lateral sclerosis (ALS) mice by activating the UPRmt to attenuate the associated neurodegenerative pathology (Zhou et al., 2020); and rapamycin, by activating mitophagy, can restore muscular endurance and improve muscular function in mitochondrial myopathy model mice (Civiletto et al., 2018). Given that mitochondrial dysfunction is a common feature of aging and chronic muscular dystrophy-associated diseases, future research efforts could look for effective targets at the level of MSRs and open new directions for therapeutic means of treatment of related diseases.
